# Incorporation of canola meal as a sustainable natural filler in PLA foams

**DOI:** 10.1186/s40643-024-00773-5

**Published:** 2024-06-05

**Authors:** Stephanie Weal, Samir Shah, Kate Parker, Alankar Vaidya

**Affiliations:** 1 Scion, Te Papa Tipu Innovation Park, 49 Sala Street, Rotorua, 3010 New Zealand; 2 35-45 Bend Road, Keysborough, VIC 3010 Australia

**Keywords:** Biocomposite, Canola meal, Foam, Natural filler, Packaging, Poly(lactic acid)

## Abstract

The canola oil industry generates significant waste as canola meal (CM) which has limited scope and applications. This study demonstrates the possibility of valorization of CM as a sustainable natural filler in a biodegradable polymer composite of Poly(lactic acid) (PLA). Generally, interfacial bonding between natural fibers and the polymer matrix in the composite is weak and non-uniform. One possible solution is to derivatize natural fibre to introduce interfacial bond strength and compatibility with the PLA polymer matrix. Here, CM was succinylated in a reactive extrusion process using succinic anhydride at 30 wt% to get 14% derivatization with 0.02 g of -COOH density per g of CM. The CM or succinylated CM at 5 and 15 wt% was co-extruded with amorphous PLA to get composite fibers. CM-PLA and succinylated CM-PLA biocomposites were foamed using a mild and green microcellular foaming process, with CO_2_ as an impregnating agent without any addition of organic solvents. The properties of the foams were analyzed using differential scanning calorimetry (DSC), Dynamic mechanical thermal analysis (DMTA), shrinkage, and imaging. The addition of CM or succinylated CM as a natural filler did not significantly change the glass transition temperature, melting point, percent crystallization, stiffness, and thermal stability of PLA foams. This suggests succinylation (modification) of CM is not a mandatory step for improving interphase compatibility with the amorphous PLA. The new PLA-CM foams can be a good alternative in the packaging industry replacing the existing petroleum-based polymer foams.

**Graphical Abstract:**

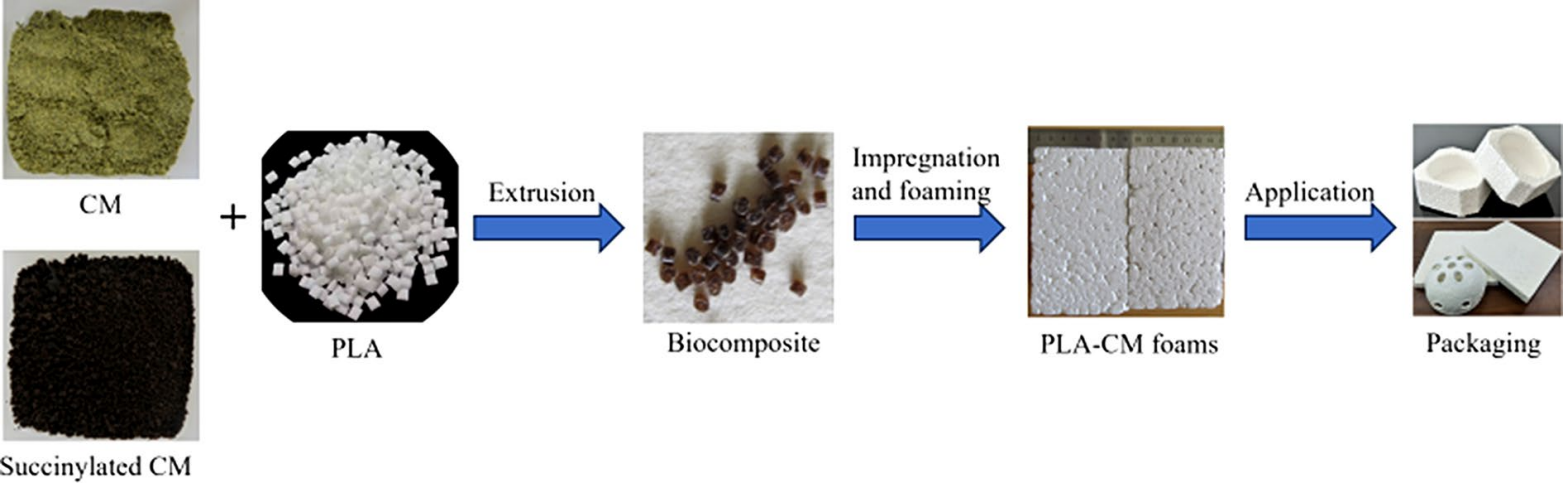

## Highlights

Valorization of CM as a sustainable natural filler in PLA biocomposite foams

Modification of CM is not mandatory for interphase compatibility with the PLA

PLA-CM foams have similar appearance and properties to control PLA foams

New PLA-CM foams can be a good alternative in the packaging industry

## Introduction

It is estimated that the utilization of plant-derived resources to produce composite materials will increase from 12% in 2010 to 25% in 2030, driven by concerns about the environment and sustainability (Wedin 2004; Li et al. 2018a). The resulting products termed biocomposites, comprise one or more phases derived from natural biological origin that can be easily disposed of or composted at the end of their life without harming the environment (de Lima et al. 2020; Manjula et al. 2017). Poly(lactic acid) (PLA; (-CH(CH3)-CO-O-)n) is the most extensively researched and utilized biodegradable thermoplastic polyester, having the potential to replace conventional fossil fuel-based polymers. PLA is a renewable (derived from potato, corn, and beet sugar), recyclable, compostable and bioabsorbable polyester that exhibits excellent processability. PLA has many industrial applications, such as in the packaging, textiles, biomedical, consumer goods and automotive industries (Gurunathan et al. 2015). However, the cost and energy consumption in PLA production and recovery are relatively high compared to synthetic polyesters such as PET (Okada 2002; Li et al. 2018b; Din et al. 2021). To reduce the cost without compromising the material properties a portion of PLA can be replaced with low-cost non-food natural materials as a filler such as flax, hemp, kenaf, henequen, banana, oil palm, jute etc. to produce PLA-based biocomposites (Dicker et al. 2014; Zini and Scandola 2011; Bourmaud et al. 2018; Siakeng et al. 2019; Hassan et al. 2023; Way et al. 2013; Zhong et al. 2011; Okubo et al. 2009; Yu et al. 2014; Jandas et al. 2012; Mysiukiewicz et al. 2022; Sun et al. 2023).

Canola (*Brassica napus L*.) is an important oilseed crop, the meal (CM) that remains after oil extraction is not used in human food or animal feed applications because of the presence of toxic compounds such as glucosinolates, phytates, erucic acid and phenolics (Manamperi et al. 2007; Li et al. 2018b; Alashi et al. 2013; Tene Tayo et al. 2022; Yang et al. 2022). Therefore, CM is an ideal resource for the replacement of synthetic polymers in developing biobased composite materials that can be applied in packaging applications. There is a strong motivation to use CM in biocomposite production (Li et al. 2018b) as CM is readily available at low cost, which will benefit the overall economics of the canola oil industry (Khattab and Arntfield 2009).

The weak interfacial bonding between polar natural fibre and non-polar organophilic polymer matrix such as PLA can compromise the properties of final biocomposites and hinder their industrial use (Yu et al. 2014; Sun et al. 2023). Various strategies have been applied to eliminate this deficiency in compatibility and interfacial bond strength, including the use of surface modification techniques (Huda et al. 2008). Chemical derivatization involves the modification of hydroxyl (- OH) and carbonyl (C = O) groups in the natural fibre introducing distinct interacting groups that can efficiently interlock with the polymer matrix at the interface (Gurunathan et al. 2015). The addition of coupling agent such as succinic anhydride (SA) provides efficient interaction with the functional surface of the natural fibre and polymer matrix. Succinylation is not selective to one type of functional group but reacts with all nucleophilic groups such as amino, phenolic, aliphatic hydroxyl, sulfhydryl, and imidazole groups. The succinyl units induce the formation of covalent bonds (via condensation reactions) and physical interactions (via hydrogen bonds) with hydroxyl groups of natural fibre thus acting as a connecting phase and providing a stronger adhesion and better mixing between the components. These interactions may allow the improvement of natural fibre and polymer matrix compatibility.

There are no literature reports on the development of PLA foams with CM or succinylated CM added as a low-cost natural filler. In this work, PLA foams were prepared with the addition of CM or succinylated CM at 5 and 15 wt% loadings and studied using differential scanning calorimetry (DSC), dynamic mechanical thermal analysis (DMTA), shrinkage and optical imaging to evaluate whether succinylation of CM is a pre-requisite step in improving interfacial bonding between CM and PLA thereby affecting the material properties of the foam.

## Materials and methods

### Materials

**Fig. 1:**
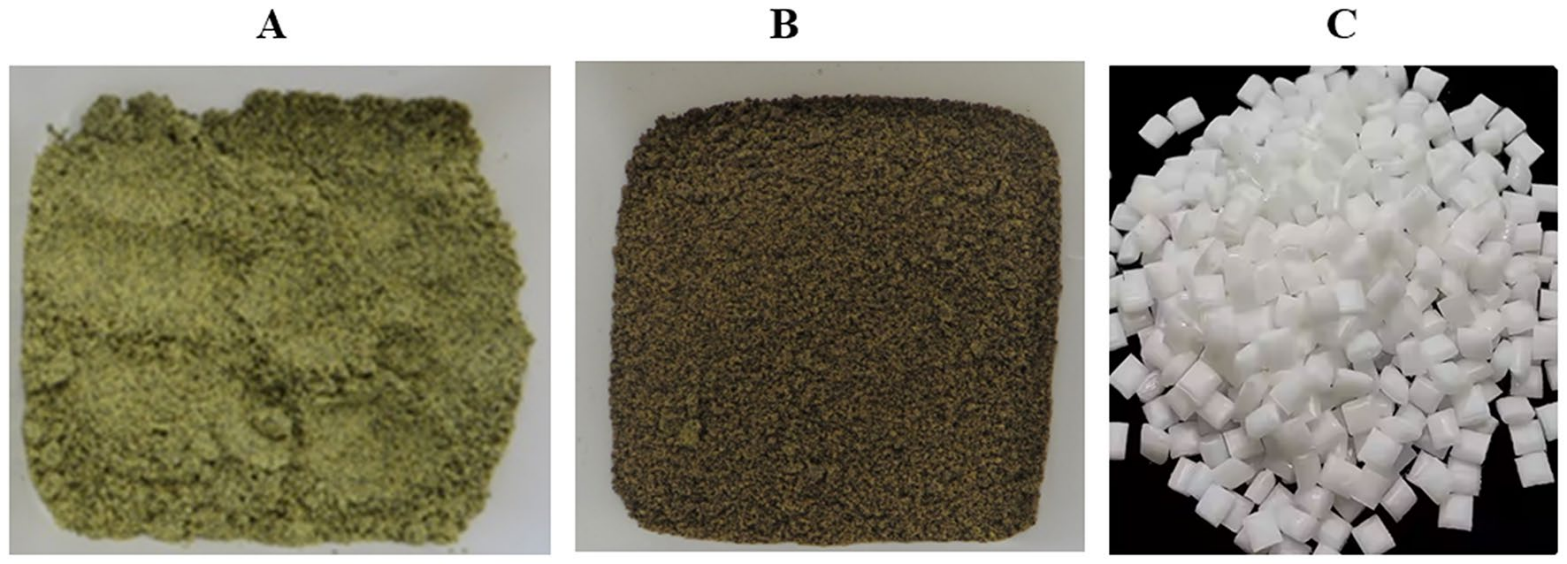
**A**) CM **B**) Succinylated CM and **C**) PLA 4060D

CM was a gift from Oil Seed Extractions Ltd. Ashburton, New Zealand. The CM is produced as a by-product from the cold pressed Rape seed oil without any solvent residues (Fig. 1). The typical composition of CM is given in Table 1. PLA Ingeo 4060D (Fig. 1), was procured from NatureWorks LLC with physical properties given in Table 1. Succinic anhydride (SA), toluene, acetone, ethanol and inorganic reagents were purchased from Aldrich, Milwaukee, USA.

**Table 1:** Composition of CM and physical properties of PLA 4060D

CM composition	% w/w
Appearance	Light yellow to green cake
Dry matter after drying at 105 ^°^C for 24 h	90
protein	34
Acid detergent fibre	17–20
Neutral detergent fibre	25
Soluble sugars	11
Oil	Not less than 12
Ash	6
Moisture	9
**PLA 4060D**	
Nature	Amorphous
D-lactic acid content	11
Density	1.24
Glass transition temperature	55–60 ^°^C
Melting point	NA

### Succinylation of CM

The dried CM was mixed with SA in a percent weight ratio of 95:5 to 50:50 respectively. This mixture was blended in a high-shear mixer at 2000 rpm for 1 min at 27 ^◦^C. Single pass extrusion without catalyst was performed at 100 g scale using a co-rotating twin screw extruder (OMC extruder, Italy) at a constant screw speed of 300 rpm (residence time ≈ 90 s). The extruder barrel temperature was set at 175 ^◦^C at least 30 min before extrusion. The extruded material was cooled down to room temperature and a 2.0 g solid was extracted in a soxlet extractor using toluene: acetone: ethanol at 4:1:1 v/v/v mixture for 18 h to remove unreacted succinic anhydride (Vaidya et al. 2016). The extracted product was dried at 60 ^◦^C for 24 h (Fig. 1).

### FTIR analysis

The FTIR spectra were recorded on Bruker model Tensor 27 at 4 cm^− 1^ resolution from 400 to 4000 cm^− 1^ and 32 scans per sample were collected (Vaidya et al. 2019). The background noise for the air and moisture was subtracted. Three replicates per sample were scanned and average data was presented.

### Acid value and degree of substitution (DS)

The acid value and DS of CM samples were determined by titration method (Vaidya et al. 2016). Briefly, two hundred milligrams of the extruded solid was suspended in 10 mL of 0.1 M NaOH at 50 ^◦^C with continuous stirring for 30 min. The excess NaOH was back-titrated with standard 0.025 M HCl in the auto-titrator (Metrohm Titrando 888) to pH 7.00. The titration was repeated three times and the average value of the HCl volume consumed was applied in the following calculations:

$${\rm{DS}}\, = \,{{{\rm{(161}} \times {{\rm{n}}_{{\rm{COOH}}}}{\rm{)}}} \over {{\rm{(m}} - 100 \times {{\rm{n}}_{{\rm{COOH}}}}{\rm{)}}}}$$

CM is made up of carbohydrates (C6 and C5 sugars), residual oil (fatty acid) and protein (amino acid). The molar mass of CM is calculated from the molar mass of a component × % wt of that component. carbohydrates (162 × 0.45 = 37.4), fatty acid (280 × 0.12 = 89.6) and amino acid (110 × 0.34 = 33.6). Therefore, mean molar mass of CM is = 37.4 + 89.6 + 33.6 = 161 g/mol (Vaidya et al. 2016), the net increase in the mass of each component unit for each succinyl unit substituted is 100 g/mol, m is the weight of the sample and n_COOH_ (acid value) is the amount of carboxyl groups calculated from the following equation:

$${{\rm{n}}_{{\rm{COOH}}}}\, = \,{{{\rm{(}}{{\rm{V}}_{{\rm{NaOH}}}} \times {{\rm{C}}_{{\rm{NaOH}}}} - {{\rm{V}}_{{\rm{HCl}}}} \times {{\rm{C}}_{{\rm{HCl}}}}{\rm{)}}} \over 2}$$

Where V is the volume and C is the concentration of HCl or NaOH.

### Preparation of biocomposite foams using PLA and CM

The CM and succinylated CM were dried at 100 ^◦^C. PLA was dried at 45 ^◦^C in an oven overnight. The formulations given in Table 2 were extruded on the 16 mm co-rotating twin screw extruder at 250 rpm. The extruder barrel temperature was set at 180 ^◦^C at least 30 min before extrusion. The PLA was fed in the first feeding port with the screw feeder. The CM or succinylated CM was manually fed in the second feeding port. After the first extrusion, the composite blends were oven-dried at 45 ^º^C overnight. All formulations were extruded a second time to ensure good mixing. The extruded strands were pelletized to get 2 mm diameter pellets.

**Table 2:** Different formulations of PLA with CM or succinylated CM in extrusion

Sample #	Formulation
1.1	PLA 4060D
2.1	95% PLA 4060D + 5% CM
2.3	85% PLA 4060D + 15% CM
3.1	95% PLA 4060D + 5% succinylated CM
3.3	85% PLA 4060D + 15% succinylated CM

The low-density PLA foams were prepared following a proprietary method (Witt and Shah 2008). Briefly, about 15–60 g of pellets were placed into a pressure vessel and impregnated with CO_2_ at 0 °C for 1 h at 60 bar. The resulting beads were placed into the freezer and weighed periodically to determine CO_2_ absorption. For pre-foaming, these beads were placed in hot water (70 °C) for 3, 5, 10, 15 and 30 s. Then beads were quenched in cold water to stop the expansion process. The traces of CO_2_ were removed from the beads by drying under circulating air for 24 h. The density of the dried beads was calculated via water displacement. These pre-foamed beads were fused into a rectangular mold by applying steam for 10 s at 90 °C followed by vacuum for 30 s. The fused rectangular foam was removed after cooling in the water.

### DSC

It was performed using a Discovery DSC (TA Instruments, USA) equipped with a refrigerated cooling system. Approximately 5 mg of fused foam sample was placed in a TZero™ aluminium pan under nitrogen (50 ml/min), heated from room temperature to 180 °C at a rate of 5 °C/min.

### Shrinkage

Approximately 57 × 57 × 20 mm size fused foam was cut, weighed, and density was calculated. The foam samples were conditioned at 23 ºC and 50% relative humidity (RH) for 48 h and placed in an oven at 60 ºC overnight. Then reconditioned under identical conditions without heating at 60 ºC before measuring the dimensions, weight and density. The percent shrinkage was calculated from the change in dimensions.

### DMTA

Dynamic mechanical properties of the quenched and fused foam samples with dimensions 50 mm × 6 mm × 2 mm were tested using a DMTA analyzer (TSA instruments, model RSAG2) in dual cantilever mode. During the tests, all samples were heated at a rate of 2 °C/min from 25 °C to 90 °C at a frequency of 1 Hz.

## Results and discussion

### Succinylation of CM

CM is regarded as a viscoelastic material yet does not exhibit classical melt flow properties like synthetic polymers e.g., PLA. The SA was selected owing to its photo-stability and fully add-on molecular skeleton in the reaction. In the extruder chemical reaction is driven by the torque and shear force of the twin screw elements. Here our objective was not to optimize the screw design or configuration but to drive solvent-free succinylation of CM.

**Fig. 2:**
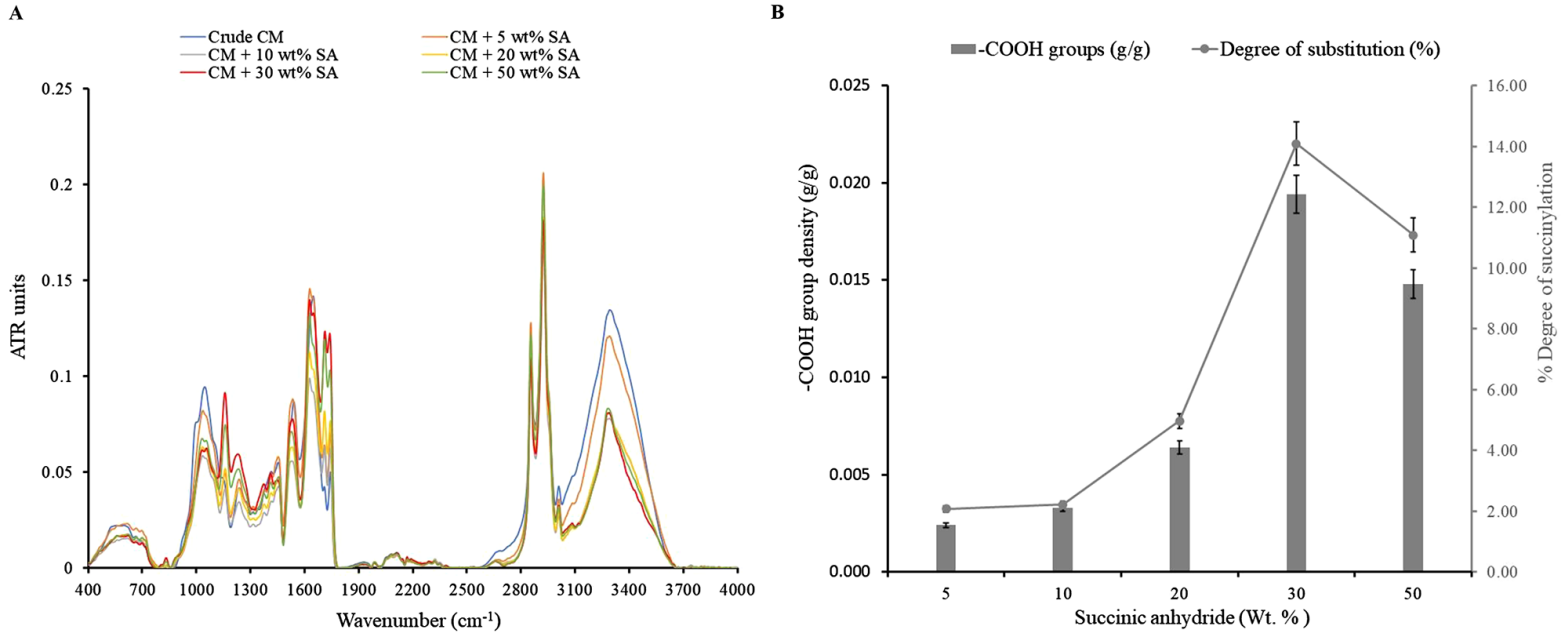
**A**) FTIR spectrum of succinylated CM; **B**) Degree of succinylation and acid value of succinylated CM. Each sample was analyzed in triplicate. The error bars denote the standard deviation of triplicate determinations

The degree of succinylation is measured as the concentration of carboxyl groups on the surface of CM (Zhao et al. 2004). At 30 wt% of SA, 14% succinylation with 0.02 g of -COOH density per g of CM was achieved (Fig. 2B). Further, an increase in the SA concentration to 50 wt% decreased the degree of succinylation and acid value. This may result from the high concentration of SA or short residence time (90 s) in the extruder. A similar result was reported in the succinylation of sander dust (Vaidya et al. 2016). This result was confirmed by FTIR as shown in Fig. 2A. The presence of 1728 and 1160 cm^− 1^ bands gave evidence for a succinylation reaction. The band at 1728 cm^− 1^ indicates absorption by carbonyl group, which is the overlap of the absorption by the ester carbonyl functionality at 1740 cm^− 1^ and anti-symmetric stretching of carboxylic groups appear at 1700 cm^− 1^ (Sun et al. 2023). Also, there were two characteristic amide bands at – amide I around 1700–1600 cm^− 1^ which overlaps with the ester carbonyl band, and amide II around 1550–1500 cm^− 1^ region corresponding to the C-N stretching band. The band at 1160 cm^− 1^ is characterized by the C-O stretching in ester and carboxylic acids. In addition, the intensity of the absorption band at 1420 cm^− 1^ is attributed to the symmetric stretching of -CH_2_ groups and -OH bending. The γ (-CH_2_) band at 830 cm^− 1^suggests the opening of the succinic ring. A strong doublet at 2900 cm^− 1^ indicates a C-H band of -CH_2_- and -CH_3_- groups of alkyl chains and a broad band at 3300 cm^− 1^ corresponds to free -OH groups and -NH stretching vibrations in the succinylated CM. These changes confirmed the succinylation of CM.

### Extrusion of PLA with CM or succinylated CM and impregnation with CO_2_

**Fig. 3:**
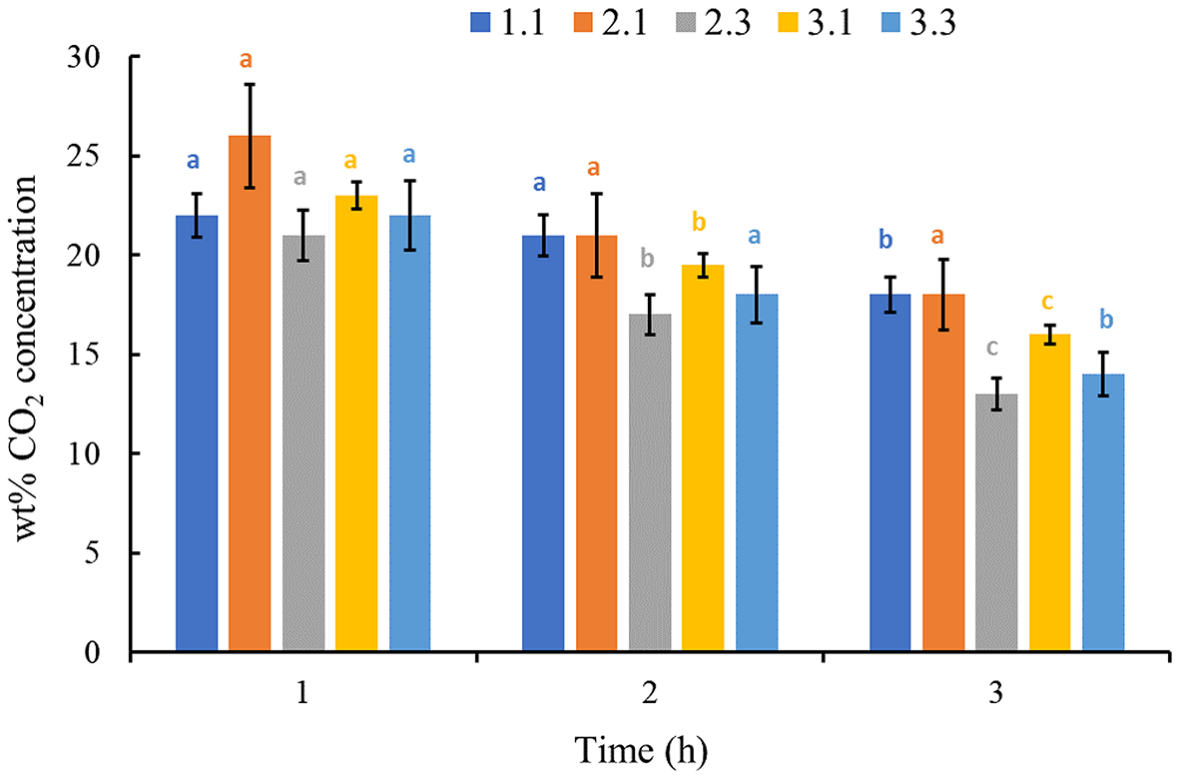
Effect of impregnation on % CO_2_ concentration in different biocomposites. The error bars denote the standard deviation of triplicate determinations. In each sample series, the colour coded data with different letters are significantly different from each other (*p* < 0.05)

PLA (95 or 85 wt%) extruded with CM or succinylated CM (5 or 15 wt%) on a co-rotating twin-screw extruder. For all formulations in Table 2, a relatively low melt pressure of 6–15 MPa, with normal torques of 30–70%, resulted in thin extruded strands that were easy to pull and pelletize. After two extrusion passes, PLA forms a continuous matrix phase and CM serves as a natural filler in the biocomposites. The CO_2_ was chosen as a gas for impregnation because it is non-toxic, non-flammable, acts as a plasticizer, is available in high purity, and has a good solubility of PLA compared to N_2_ (Villamil Jiménez et al. 2024). Further, CO_2_ is a green solvent that can be completely removed at ambient conditions from the foam (Milovanovic et al. 2023). Typically, at the start of the impregnation around 22 wt% of CO_2_ was absorbed by all formulations except sample # 2.1 (Fig. 3). For control PLA polymer after 3 h of impregnation, the absorbed CO_2_ drops down to 18 wt% which is the ideal concentration required for pre-foaming and moulding. After 3 h of impregnation, the composite samples containing CM or succinylated CM demonstrate a reduced wt% of CO_2_ concentration than the control PLA sample. This could be due to the heterogeneous CM particles thermodynamically immiscible with the PLA matrix creating channels that allow diffusion of CO_2_ compared to the more homogeneous and compact control PLA sample. However, in the case of sample # 3.3 with a higher percent of succinylated CM retains 15 wt% of CO_2_ compared to 13 wt% CO_2_ retention in sample # 2.3 having 15 wt% CM. This suggests addition of 15 wt% of succinylated CM in the PLA biocomposite marginally decreases diffusional loss of CO_2_ due to the affinity of succinyl groups towards CO_2_ (de Teixeira et al. 2014; Han and Ho 2018).

### Pre-foaming of the extruded PLA with CM or succinylated CM

**Fig. 4:**
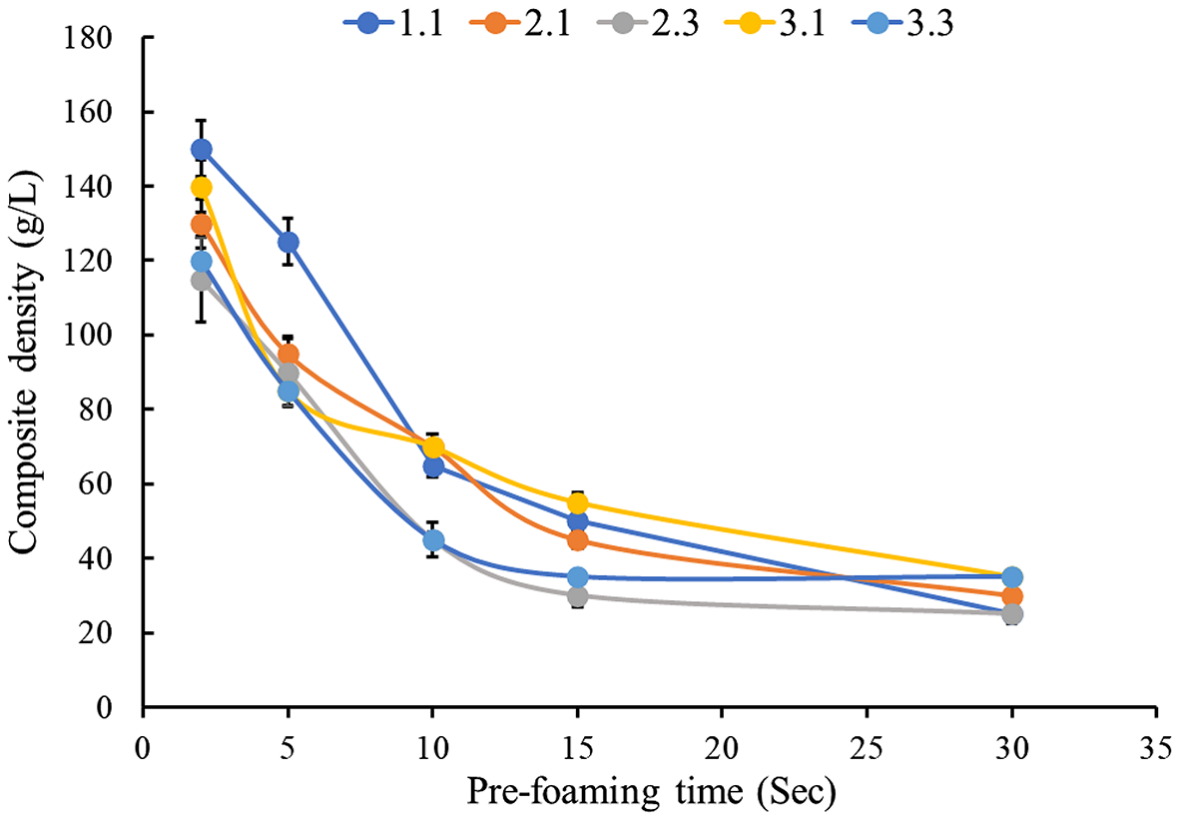
Change in densities of biocomposites during pre-foaming relative to pre-foaming time. The error bars denote the standard deviation of triplicate determinations

The dissolved CO_2_ molecules increase the intermolecular distance causing PLA polymer to swell, increasing specific volume, and decreasing the overall density of all pre-foamed PLA formulations (Milovanovic et al. 2023). After 3 s of pre-foaming, the densities were in the range of 110–150 g/L, and after 30 s densities reached lower than 50 g/L (Fig. 4). In the initial stages of pre-foaming PLA-4060D is amorphous, but during pre-foaming the PLA polymer chains align parallel to each other exhibiting semi-crystalline behaviour. The solubility of CO_2_ in semi-crystalline PLA is more complex than amorphous PLA because CO_2_ can accelerate the crystallization process. Moreover, the presence of crystals decreases the CO_2_ solubility in the PLA matrix and dramatically affects cell nucleation and cell growth. All these factors together can decrease the overall densities of pre-foam beads (Nofar and Park 2014).

In the first 15 s of pre-foaming the decrease in density was rapid, especially for samples containing 15 wt% CM (sample # 2.3) or succinylated CM (sample # 3.3) compared to the control PLA (sample # 1.1) or 5 wt% CM (sample # 2.1) or succinylated CM (sample # 3.1). As mentioned earlier, the presence of a higher proportion of CM provides greater affinity for CO_2_ molecules which in turn provides more interactions of CO_2_ with PLA beads that leads to rapid pre-foaming observed in the first 15 s. Further, during prefoaming a pressure drop, or a temperature increase can cause cell nucleation and growth due to the thermodynamic instability of the gaseous CO_2_. However, at 30 s of prefoaming all samples showed similar densities in the range of 25–40 g/L. This indicates foam stabilization after 30 s of prefoaming with complete removal of CO_2_ from the foam.

### Fusing pre-foamed PLA with CM or succinylated CM

**Fig. 5:**
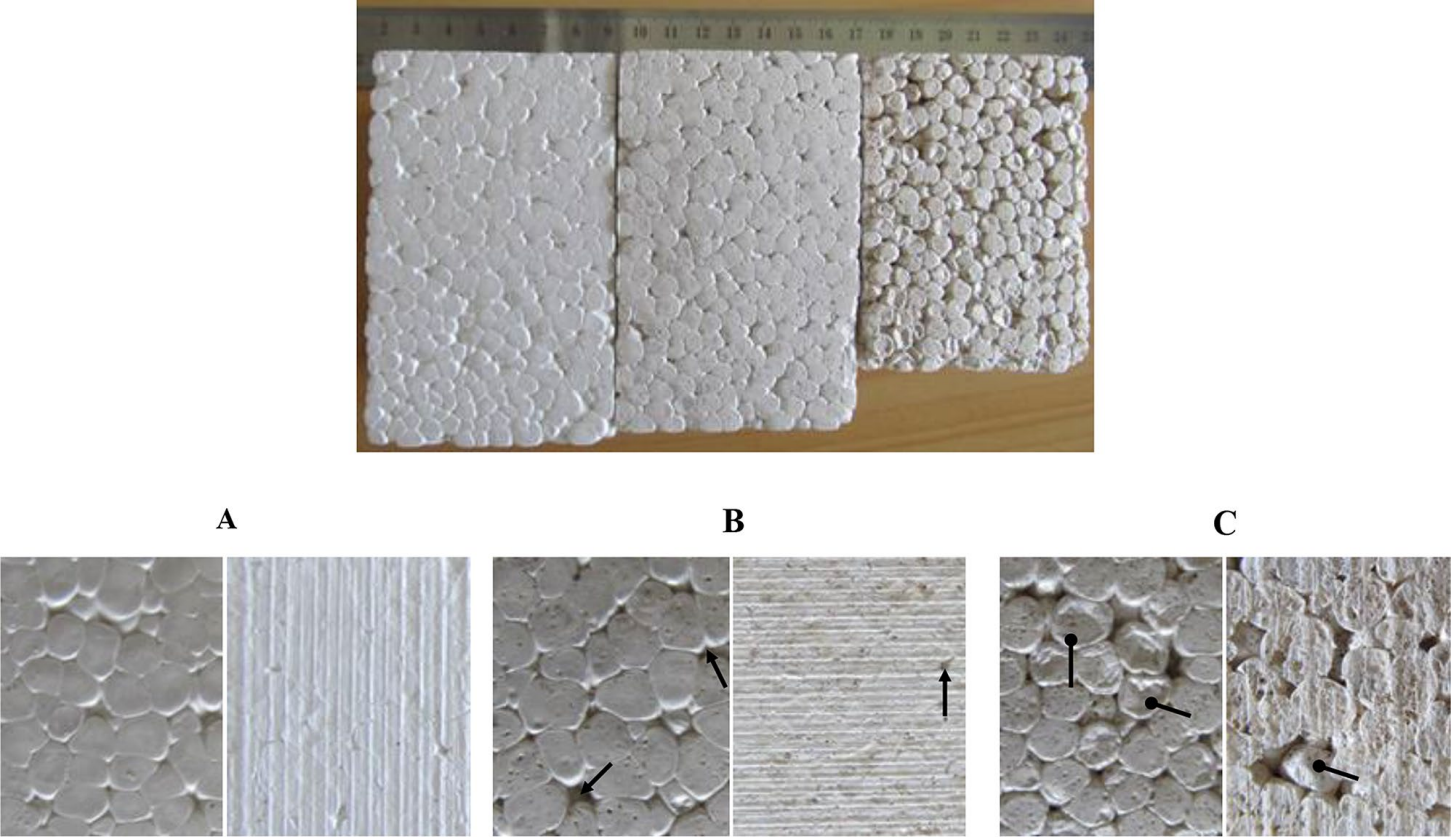
Top row - fused foam blocks, Left -control PLA; middle – with 5 wt% CM, and right – with 15wt% CM. Bottom row - optical images, **A**) Control PLA; **B**) with 5 wt% CM; **C**) with 15 wt% CM. The left are normal views, and the right images are cut surface views

In temperature-induced foaming, thermodynamic instability is caused by the immersion of pre-foamed PLA polymer with CO_2_ under elevated pressure in hot water. Foaming is initiated when the applied temperature is increased which leads to enhanced PLA chain mobility as the polymer is softened and CO_2_ solubility decreases, which results in cell nucleation and growth. The final cooling step ensures the stabilization of the foam (Standau et al. 2019). Note the imaging results of PLA fused with CM are only discussed since PLA with succinylated CM foams have identical features. The control PLA foam was white while, the addition of 5 wt% CM, gave a slightly brown coloration that further darkens with the addition of 15 wt% CM (Fig. 5 top row).

On close inspection of the foam surface, it is revealed that control PLA foam is smooth with beads uniformly expanded without leaving conspicuous voids or gaps, this was further evident from the cut surface showing the fine cell structure with all beads expanded to fill the available space. With the addition of 5 wt% CM in the foam clearly showed pits (arrow in Fig. 5B) due to the presence of individual CM particles and the cut surface showing micro-pores with coloured CM patches. Further increase in CM loading to 15 wt%, resulted in more pits and bead instability (oval arrow in Fig. 5C). The cut surface shows a coarse cell structure with pores due to uneven expansion of the beads not able to fill the gaps. A similar observation was reported by Sun et al. (2023) with an increase in the rice husk from 5 to 30 wt% in the PLA-maleic anhydride copolymer more uneven and large cavities are produced in the foams due to rice fibre agglomeration reducing the surface adhesion with PLA matrix. Comparable, micro-sized pores in the open cell structure were reported in PLA/rHDPE/kenaf fibre composites (Hassan et al. 2023). Moreover, the major toxic compounds present in CM such as glucosinolates (decomposition at 99 ^o^C) and phytates (decomposition at 150 ^o^C) are heat labile and degrade at the extrusion temperature of 175–180 ^o^C. The PLA-CM foams can safely be used in non-food contact packaging applications.

### Thermo-mechanical properties of the foams

**Fig. 6:**
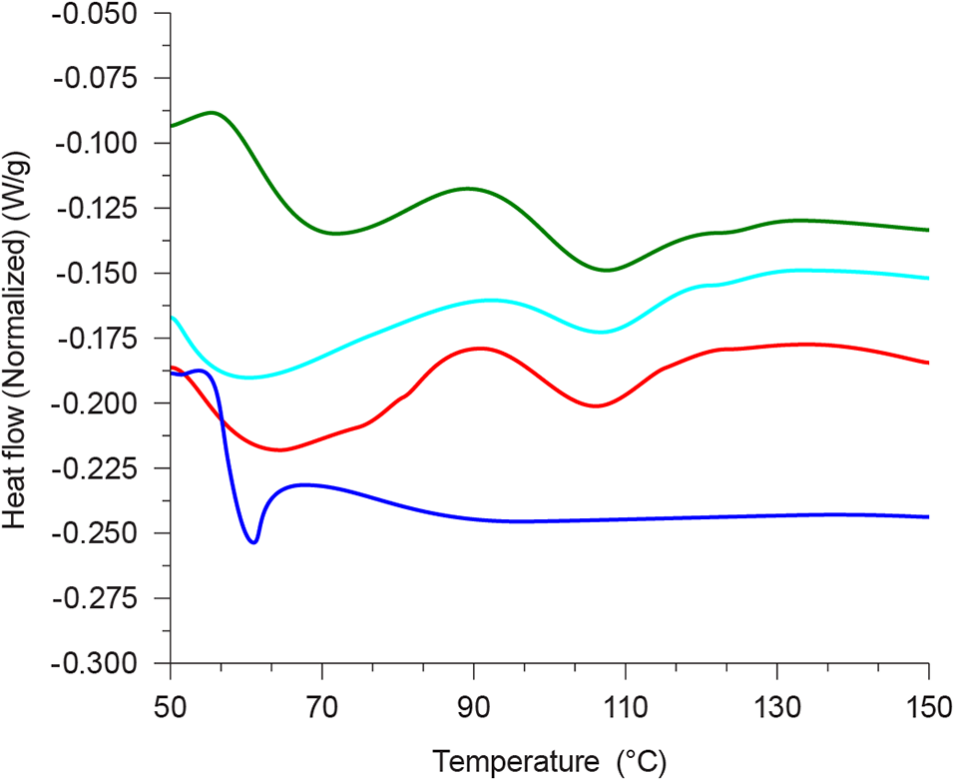
DSC of PLA foams. blue - control PLA before foaming; green - control PLA after foaming; red - PLA + 15 wt% CM; teal – PLA + 15 wt% succinylated CM

The DSC scan of the control-foamed PLA was typical for an amorphous polymer, showing a glass transition temperature (T_g_) at 58 ^º^C with no observed melting peak (Fig. 6). On foaming PLA shows a DSC scan for a semi-crystalline polymer with a slightly higher T_g_ (60 ^º^C) followed by an endothermic relaxation often attributed to a more organised secondary molecular rearrangement in the amorphous phase of the polymer (Villamil Jiménez et al. 2024). The percent crystallinity of the foamed PLA was calculated to be 4.5% based on the heat of fusion of the pure PLA = 93 J/g (Yilmaz et al. 2024). Also, a broad melting peak at around 108 ^º^C was observed. The addition of 15 wt% CM to the PLA did not significantly change the T_g_, melting point or percent crystallization. A similar observation was reported by Villamil Jiménez et al. (2024) foaming PLA with 5 or 15 wt% cellulose. However, with the addition of 15 wt% of succinylated CM, T_g_ (52 °C) and melting point (105 °C) were slightly reduced compared to control PLA foam. This can be due to the interaction of succinyl groups with the PLA polymer chains causing disorganized packing in the semi-crystalline PLA polymers.

**Fig. 7:**
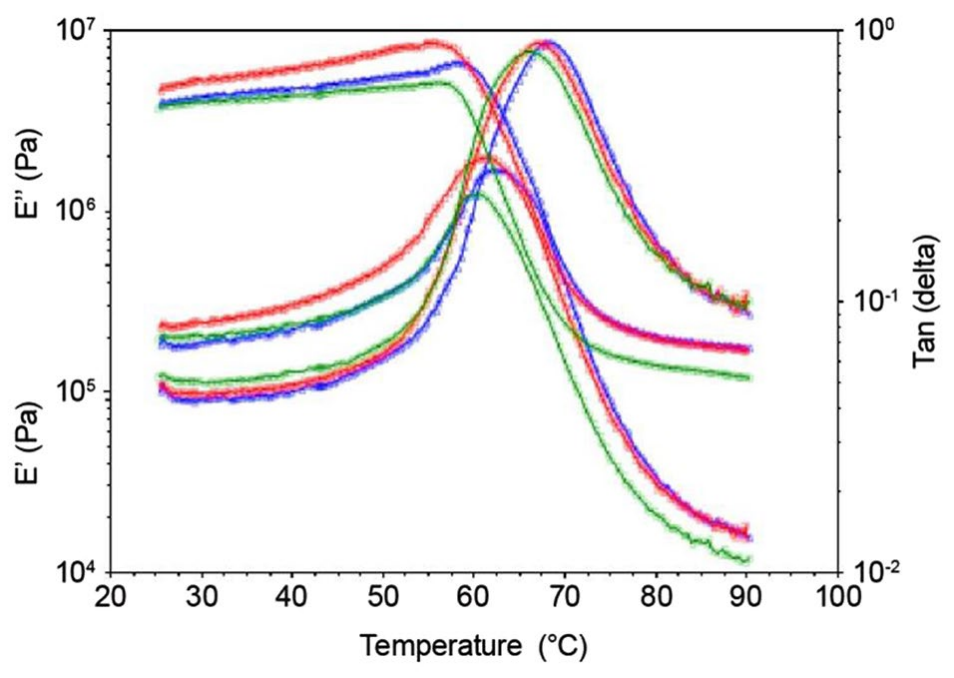
DMTA of PLA + CM foams. Blue - control PLA foam, red - PLA + 5 wt% CM, green - PLA + 15 wt% CM

DMTA was used to determine the storage and loss moduli of the foam blocks. Storage modulus (E’) measures the stiffness of the material, a higher E’ indicates a stiffer material. For the control PLA foam, the E’ rapidly decreases at around 60 ºC (T_g_), indicating the heat deflection temperature (HDT) of the foam (Fig. 7). The HDT is the short-term heat stability of the foam. For semi-crystalline polymers like PLA foam, the HDT is increased by increasing percent crystallinity. The loss modulus (E”) of the control PLA foam peaks at 60 ºC (T_g_). The PLA + 5 wt% CM foam has a similar DMTA scan as the control PLA foam, with a slightly higher storage modulus indicating a stiffer foam. For the PLA + 15 wt% CM foam, the storage modulus rapidly decreases at 57 °C, which is a lower temperature compared to control PLA foam and PLA + 5 wt% CM foam. A similar observation for loss modulus (E”) shows a peak at 58 °C. This suggests the addition of 15 wt% CM did not change appreciably the thermal stability. Thus, DMTA data correlates with the DSC results.

### Shrinkage

After 16 h at 60 °C, all PLA foams shrunk by about ~ 50% or increased in density by almost 100%. This temperature is just above the T_g_ of PLA foams allowing the PLA polymer chains to become mobile and restore the PLA polymer to their native amorphous state. The addition of CM or succinylated CM has not changed shrinkage stability significantly.

## Conclusions

A sustainable waste product (CM) was succinylated in a reactive extrusion process. PLA was extruded with CM or succinylated CM on a co-rotating twin-screw extruder to prepare a biocomposite material. The biocomposite foam was prepared using microcellular foaming technology with CO_2_ as an impregnating agent without any addition of organic solvents. The control PLA foam is white, smooth and beads uniformly expanded without conspicuous voids or gaps whereas, the addition of CM as a natural filler in the PLA foam, resulted in a slightly brownish foam with some pits and micro-voids. The material properties of the foams confirmed succinylation of CM is not a pre-requisite step for improving inter-phase compatibility between PLA and CM. The addition of CM to the PLA foam did not significantly change the T_g_, melting point, percent crystallization, stiffness, and thermal stability. The newly developed PLA-CM foams can be used in non-food packaging applications providing a valuable option to replace petroleum-based non-biodegradable foams.

## Data Availability

The datasets used and/or analyzed during this study are available upon reasonable request.
